# Monthly Summary

**Published:** 1884-05

**Authors:** 


### MONTHLY SUMMARY.

---

THE THERAPEUTIC APPLICATION OF NITROUS OXIDE GAS. —From a series of experiments made in Prof. Botkin's laboratory in St. Petersburg, Dr. S. Klikowitsch (*Virchow's Archiv.* xliv., 2) draws the following conclusions:

1. Nitrous oxide gas is incapable of supporting respiration in animals and plants, and, like other indifferent gases, leads to death from asphyxia. The asphyxia produced by this gas, however, presents points of contrast to the asphyxia produced by other means.

2. Nitrous oxide gas produces no chemical or morphological changes in the blood of animals, but is dissolved in it and again eliminated, according to physical laws, without apparently being broken up into nitrogen and oxygen.

3. Anæsthesia with laughing gas is closely associated with insufficient oxidation of the blood that it cannot be regarded as absolutely without danger, especially in diseases of the heart, lungs or blood vessels.

4. The association of laughing gas with twenty per cent. of oxygen completely removes the possibility of asphyxia, and produces a number of results capable of therapeutic application.

5. Under the influence of the mixture of laughing gas and twenty per cent. oxygen, in the majority of healthy subjects, the heart's pulsations are increased, the pulse-wave diminished, and the respiratory movements decreased in number and increased in depth; these effects pass off in from three to five minutes.

6. In six weeks of weak heart-action, the above gaseous mixture produced no unfavorable results; on the other hand, the pulse was decreased in frequency and increased in strength. These effects lasted from one to two hours.

7. In cases of disturbed respiratory innervation the mixture of laughing gas and oxygen regulated the respiratory rhythm and rapidly removed the subjective and objective signs of deficient oxidation of the blood.

8. This gaseous mixture acts as a transient anæsthetic, and in angina pectoris causes a rapid removal of suffering.

9. It is to be preferred to chloroform as an anæsthetic in labor.

10. Vomiting and cough of reflex origin are arrested by a few inhalations of this mixture of gases.—*Philadelphia Medical Times.*

ARTIFICIAL CROWNS.—We have made some very serviceable artificial crowns on bicuspid roots in the following manner: Trim up the end of the root neatly, and drill a tolerably good sized hole into it in a direct line, Into this, carefully but firmly, introduce an ordinary steel screw, such as can be procured at the hardware store. Fit around the root a cage or cell of annealed, thin saw blade such as we use for other purposes, and of such a length as to come to a line with the required height of the crown. When adjusted, tie it with fine wire; or, if there is not sufficient space between the teeth, secure with waxed silk floss. Then mix a sufficient quantity of amalgam *very dry* and *mallet* it into the cage and around the screw with tolerably warm instruments, The patient may then be dismissed for two or three or four hours, with the cage *in situ*, keeping a thin disc of cork between some of the teeth to prevent contact for the time being. Upon returning the cage is removed, and a few minutes with the file, corundum stone, and emery cloth make a good finish. The operation is speedily performed, is inexpensive. and makes a good masticator. The *dry* amalgam contains less mercury, and when malleted with warm instruments becomes hard in an hour or two, sometimes a little longer. The rubber dam may be used if there is sufficient projection of the root; but we do not think it necessary, the napkin keeping things dry long enough to pack in the amalgam.—*Dental Register.*

---

ETHER.—In the *Lancet*, Dr. H. Bendelack Hewetson discusses the relative value of ether when prepared with "rectified" or "methylated" spirits of wine. The author states that there is a great difference between them. That prepared with rectified spirits has been found by those who have used it less safe and less desirable, producing more sickness and laryngeal spasm in certain cases in which there is a tendency to such complications. Of the use and applicability of the methylated ether—as the safest anæsthetic known, when carefully administered by means of Clover's inhaler—he can speak strongly, as the result of daily observation. It is a very ordinary circumstance to occupy eighty seconds in producing complete anæsthesia, without a struggle or a cough, and it is by no means extraordinary for a patient to be "fully under" *within* the minute. In the case of short operations upon the eyes, and the like, it is hardly ever necessary to reapply the inhaler after it has once removed for the operator to commence, the patient remaining sufficiently anæsthetic for an operation such as mentioned to be completed without hurry. Anæsthesia can be prolonged with equal safety even so far as to keep a patient in labor completely under its influence for upwards of *four hours*, the longest time which has happened in my experience. Methylated ether is, I consider, from this point of view, the safest and cheapest anæsthetic at present in use.—*Medical and Surgical Reporter.*

